# Quality of Life of Vegetarians during the COVID-19 Pandemic in Brazil

**DOI:** 10.3390/nu13082651

**Published:** 2021-07-30

**Authors:** Shila Minari Hargreaves, Eduardo Yoshio Nakano, Heesup Han, António Raposo, Antonio Ariza-Montes, Alejandro Vega-Muñoz, Renata Puppin Zandonadi

**Affiliations:** 1 Department of Nutrition, Faculty of Health Sciences, Campus Darcy Ribeiro, University of Brasilia (UnB), Asa Norte, Brasilia 70910-900, DF, Brazil; shilaminari@gmail.com (S.M.H.); renatapz@unb.br (R.P.Z.); 2 Department of Statistics, University of Brasilia, Brasilia 70910-900, DF, Brazil; eynakano@gmail.com; 3 College of Hospitality and Tourism Management, Sejong University, 98 Gunja-Dong, Gwanjin-Gu, Seoul 143-747, Korea; 4 CBIOS (Research Center for Biosciences and Health Technologies), Universidade Lusófona de Humanidades e Tecnologias, Campo Grande 376, 1749-024 Lisboa, Portugal; 5 Social Matters Research Group, Universidad Loyola Andalucía, C/Escritor Castilla Aguayo, 4, 14004 Córdoba, Spain; ariza@uloyola.es; 6 Public Policy Observatory, Universidad Autónoma de Chile, Santiago 7500912, Chile; alejandro.vega@uautonoma.cl

**Keywords:** COVID-19, pandemic, vegetarians, quality of life

## Abstract

Health emergencies such as the COVID-19 pandemic can negatively impact quality of life (QoL) due to higher levels of stress, social isolation, and uncertainties. In this scenario, distinct population groups might react differently. Vegetarians, who follow a non-conventional dietary pattern, could be more vulnerable to the abrupt changes in normal life routine and economic instability. Therefore, this study aimed at evaluating if the current pandemic situation somehow affected vegetarians’ QoL. A cross-sectional study was carried out in Brazil between 28 July and 14 September 2020 to evaluate the QoL in vegetarians during the pandemic period. Vegetarian adults replied to an online survey that included the VEGQOL and WHOQOL-BREF instruments to evaluate QoL and questions related to the COVID-19 pandemic. A total of 1282 individuals participated. Only 3.8% had tested positive for COVID-19, but 39.9% affirmed having a family member who tested positive for the disease. Almost half (46.3%) of the sample had an income drop due to the pandemic. Results of QoL scores in the different subcategories of vegetarians were similar to previously published data. Individuals who had already tested positive for COVID-19 had lower QoL scores than those who did not test positive, but only in the VEGQOL. QoL was lower for the participants who declared that Sars-Cov-2 had already infected a family member for almost all the parameters evaluated. On the other hand, an income drop affected QoL only partially. Studying how vegetarians are influenced by such conditions contributes to the generation of relevant data that can be used to support healthcare and public policies in the future.

## 1. Introduction

The Coronavirus 19 disease (COVID-19) is the disease caused by the severe acute respiratory syndrome virus 2 (SARS-CoV-2), identified in January 2020 as the cause of the respiratory syndrome epidemic affecting the city of Wuhan, in China. Due to its high contagious rate, the disease soon spread worldwide, being declared a public health emergency of international concern by the World Health Organization (WHO) on 30 January 2020 [1]. As of July 2021, over 190 million cases of COVID-19 had already been confirmed, with 4,093,145 deaths worldwide. In Brazil, the number of deaths reached 541,266, representing 13.2% of all deaths from COVID-19 in the World, the second-highest number, surpassed only by the United States of America, with 603,790 deaths (numbers updated on 19 July 2021) [2].

Health emergencies can negatively affect mental health and quality of life, due to fear-related higher level of stress and anxiety, social isolation, and misinformation. In Italy, the first European country to face the a COVID-19 pandemic and implement lockdown measures, a study conducted between March and August 2020 found high rates of negative mental health outcomes, which included: post-traumatic stress symptoms, depression, anxiety, insomnia, perceived stress, and adjustment disorder [3]. The impact of the COVID-19 pandemic was also evaluated in Liaoning a province in China. The pandemic was associated with mildly stressful impacts, with 52.1% of the participants reporting feeling horrified and apprehensive due to the situation [4].

Quality of life is a subjective concept that encompasses different dimensions: physical, psychological, social, and spiritual [5]. It relates to the quality of the environment in which the individual lives; their mental and physical health; the feeling of “utility”, which means feeling useful for society; and the appreciation and satisfaction with life [6]. It is known that adopting different dietary patterns can influence one’s quality of life [7], mainly during a pandemic period due to the fear of limited access to food [8]. A recent study conducted in Poland with 1033 adults showed that perceived stress is a predictor of consumers’ fear of limited access to food and a predictor of food-purchase behaviors during the Sars-Cov-2 pandemic [8]. The study showed that reduced food supplies in grocery stores were noted by more than half of the participants, who attributed this to the pandemic period. Experiencing fear during a pandemic, which is caused mainly by changes in food availability, raises the question of the importance of available information and trust in their sources in reducing this negative emotion and its consequences [8]. Therefore, we hypothesized that the Sars-Cov-2 pandemic could have a particular impact on vegetarians, affecting their quality of life. Despite being seen as a restrictive dietary pattern, a vegetarian diet did not seem to negatively affect the quality of life before the pandemic period [9,10,11,12].

Following a vegetarian diet many impact an individual’s routine of buying food, preparing meals, and eating out, since most of the population are not vegetarian and, therefore, vegetarian options might be limited in groceries stores and restaurants. It has already been demonstrated that vegetarians (especially vegans) in Brazil have good quality of life [13]. However, no study evaluating the quality of life in vegetarians during a period of major stress, social restrictions, limited access to in-person purchases, and economic instability has yet been published. We hypothesize that this situation could negatively affect the quality of life, especially for those who had family members testing positive for the virus, who were infected themselves, or whose economic situation worsened. Therefore, this study aimed at evaluating if the current pandemic situation somehow affected vegetarians’ quality of life.

## 2. Materials and Methods

### 2.1. Study Design

A cross-sectional study was carried out in Brazil by an online survey to evaluate the quality of life in vegetarians during the pandemic. Individuals who classified themselves as vegetarians were invited to participate through messaging and social media advertisement. The study was conducted from 28 July to 14 September 2020, when the consequences of over four months of imposed social isolation and circulation restrictions could be evaluated in this population group.

### 2.2. Variables and Instruments

To measure the quality of life, two different questionnaires were used: the Quality of Life Questionnaire for Vegetarians (VEGQOL) [13] and the short version of the Quality of Life Questionnaire from the World Health Organization (WHOQOL-BREF) [14]. Sociodemographic data and self-referred anthropometric data to calculate body mass index (BMI) were also collected. Moreover, questions related to the COVID-19 pandemic were included to evaluate if participants or their relatives had had the disease, and if the global crisis had affected their economic situation due to income reduction/loss.

The VEGQOL is a specific tool developed to evaluate the quality of life of vegetarians. Its original version was developed in the Brazilian-Portuguese language and validated for the Brazilian vegetarian population [13]. It includes (besides the questions related to quality of life) items to categorize the vegetarian population. Firstly, vegetarians are classified into different types of diet. For this purpose, four different categories are considered: flexitarian (includes meat no more than once per week or excludes only red meat); pescatarian (excludes all types of meats except for fish); ovolacto-vegetarian (excludes all types of meats but includes eggs and/or dairy products); and vegan (excludes all foods from animal origin). Moreover, the motivation to adopt a vegetarian diet is also considered. The main reasons included in the questionnaire were: ethical/moral reasons, personal health, religion/spirituality/beliefs, environmental impact, and aversion/intolerance. VEGQOL also includes an item “other reasons” for the individuals who did not identify themselves with any of those categories. Time adopting a vegetarian diet and having close people also adopting it were also included as variables [13].

The WHOQOL-BREF was developed to evaluate general QoL, and it is composed of 26 items, of which 24 are divided into four different domains: physical health (domain 1), psychological wellbeing (domain 2), social relationships (domain 3), and environment (domain 4), plus two general items that are analyzed separately. Domain 1 comprises aspects related to pain and discomfort; sleep and rest; energy and fatigue; mobility; activities of daily living; dependence on medical substances and medical aids; and work capacity. Domain 2 encompasses the following facets: positive feelings; thinking, learning, memory and concentration; self-esteem; bodily image and appearance; negative feelings; and spirituality/religion/personal beliefs. The components of domain 3 are: personal relationships; social support; and sexual activity. Domain 4 comprises freedom, physical safety and security; home environment; financial resources; health and social care: accessibility and quality; opportunities for acquiring new information and skills; participation in and opportunities for recreation/leisure activity; physical environment (pollution/noise/traffic/climate); and transport [14]. The remaining two items from WHOQOL-BREF are: (1) “How would you rate your quality of life?”; and (2) “How satisfied are you with your health?”, and are analyzed separately [15].

### 2.3. Subjects

Vegetarian adults (18 years old or above) from the entire country were invited to participate. The invitation was spread through social-media advertising and messaging. Individuals who accepted the invitation had access to a link that led to the SurveyMonkey^®^ platform, on which the research instrument was built. Participants were only directed to the questionnaire after accepting a consent form. Only fully answered questionnaires were used, others complete were excluded from the analysis. This study was approved by the Ethics Committee University of Brasília’s Health Institute (protocol number: 94114118.7.0000.0030) and conducted according to the guidelines laid down in the Declaration of Helsinki.

### 2.4. Data Analysis

Data from the VEGQOL were analyzed as described by Hargreaves et at. [13]. The items were evaluated using a five-point Likert scale for each. The sum of all items generates a score, and then it was converted to a 100-point scale. The higher the score, the better one’s quality of life is. WHOQOL-BREF, on the other hand, is analyzed by each one of its four domains separately. Scores for each domain (physical health, psychological wellbeing, social relations, and environment) result in a scale ranging from 1 to 5. From the 26 items that compose WHOQOL-BREF, 24 integrate the four domains, and the other two are analyzed separately, being used as indicators of overall quality of life [14,16]. Variables related to vegetarianism (type of diet, motivation, time adopting the diet, and close people also adopting the diet) and to the COVID-19 pandemic (individual or relative testing positive for COVID-19, and income reduction) were used to evaluate the impact of the pandemic on vegetarian’s quality of life. Differences between scores for each variable related to vegetarianism and COVID-19 pandemic were examined with independent Student t-test Anova with Tukey’s post-hoc tests, Mann-Whitney U-test, and Kruskall-Walis test with Bonferroni’s post-hoc tests. The level of statistical significance was set at 5% (*p* < 0.05). The statistical software IBM SPSS Statistics for Windows (Armonk, NY: IBM Corp) was used to conduct the analyses.

## 3. Results

### 3.1. Sociodemographic Data

A total of 1547 individuals entered the survey, of which 1282 answered the questionnaire until the end, composing the final sample of the research, distributed among all five Brazilian regions. Table 1 describes the characteristics of the studied sample. Only 3.8% of the individuals reported having tested positive for COVID-19, but 39.9% affirmed having a family member who tested positive for the disease. From those, most of them (32.6%) reported not living together with the family member who tested positive. Almost half (46.3%) of the sample had an income drop due to the pandemic.

### 3.2. Quality of Life: VEGQOL

Individuals who had already tested positive for COVID-19 had lower QoL scores (71.51 ± 13.02) than those who did not test positive (75.45 ± 11.43). A significantly lower score was also observed when a family member had been infected by COVID-19 (*p* = 0.016). The scores were 73.64 ± 12.21 when the family lived together and 74.30 ± 11.87 when they did not live together. No statistical difference was observed among those who did have or did not have a reduction in their average income (75.23 ± 12.24 and 75.38 ± 10.84, respectively; *p* = 0.827) (Table 2). No differences in QoL scores were observed in terms of among age, gender, average income, housing location, and educational level (Table 2). Normal weight individuals (BMI: 18.5 to 24.9 kg/m^2^) had statistically higher QoL average score (75.83 ± 11.31) than the overweight ones (BMI > 24.9 kg/m^2^) (73.73 ± 11.99). Individuals with low BMI (<18.5 kg/m^2^) had intermediate results (75.83 ± 11.11) with no statistical difference from the other two groups.

Among the different types of vegetarians, vegans had a better QoL score (80.70 ±10.07). In fact, only vegans had an average QoL considered “high”, according to the VEGQOL cut-off points [13]. Ovolacto-vegetarians had an average score of 75.00 ± 10.37, followed by pescatarians (70.46 ± 11.57), both classified as having “satisfactory” QoL [13]. Flexitarians had the lowest average score (66.43 ± 11.86), considered “regular”. The participants who adopted a vegetarian diet for a shorter time (less than one year) showed worse QoL (71.54 ± 12.04) than the ones who had been following the diet from one to five years, and more than five years (76.41 ± 10.85 and 77.75 ± 11.07, respectively).

Regarding the different motivations for adopting a vegetarian diet, individuals who adopted it for ethical/moral reasons had the highest average score (76.40 ± 11.24). The lowest scores were observed for those who adopted the diet due to environmental impact (73.28 ± 11.64) and aversion/intolerance (71.60 ± 13.33). Intermediate scores were found among the ones adopting a vegetarian diet for personal health (74.64 ± 11.33), religion/beliefs (74.95 ± 12.29) and other reasons (75.63 ± 11.68). Moreover, the participants who had close people also adopt a vegetarian diet had the higher QoL score (76.45 ± 10.86) over those who did not (72.02 ± 12.62).

### 3.3. Quality of Life: WHOQOL-BREF

WHOQOL-BREF first two items ([1] “How would you rate your quality of life?”; and [2] “How satisfied are you with your health?”) are analyzed separately and used to evaluate the overall quality of life and health satisfaction. Considering the entire sample, the scores’ values for items 1 and 2 were 4.26 ± 0.67 and 3.87 ± 0.87, respectively. Scores for item 1 were lower in overweight individuals when compared to those with normal weight, with underweight participants showing intermediate scores. A higher income also correlated with better scores for this item, and income reductions negatively affected the results. Regarding the type of diet, scores for item 1 were higher for vegans and pescatarians, and lower for flexitarians. A better score was also found among the individuals who had close people also following a vegetarian diet. Score trends for item 2 were very similar to those for item 1, except for the last variable (close people also adopting a vegetarian diet), in which no statistical difference was found. For item 2, vegans had higher scores, with no difference between ovolacto-vegetarians and pescatarians (intermediate values), and lower scores for flexitarians. In addition, results for item 2 were worse among individuals who had a family member infected by COVID-19 (with statistically different results only when the family member did not live together with the person).

Results for each of the four different WHOQOL-BREF domains are described in Table 2. Scores for domain 1 (physical health) were lower for individuals who reported having a family member (not living together) infected by COVID-19. Females had lower scores than males and, similarly to the results for items 1 and 2, overweight people had lower scores than normal-weight individuals, with intermediate values for underweight participants. Physical health scores were also better for those with a higher educational level and higher average income, with worse results for those receiving less than two minimum wages, and better scores for those who reported receiving more than 20 minimum wages. The other income ranges had intermediate score results. Having an income reduction during the pandemic resulted in lower scores for the participants. Physical domain scores were also higher for vegans and lower for flexitarians. Regarding different motivations to adopt a vegetarian diet, the ones who adopted it due to aversion/intolerance had lower scores for the physical domain, and the ones who marked “others” as a motivation had higher scores, but with no statistical difference to all other categories, except for “aversion/intolerance”. Scores were also higher for participants who had close people also adopting a vegetarian diet.

In domain 2 (psychological wellbeing), the average score was also lower for the ones having a family member infected by COVID-19 (not living together). Females scored lower, and in this domain, younger people (below 40 years old) also had lower scores. Correlation between domain 2 scores and BMI, as well as for the educational level, were the same as in domain 1. Only participants with an income lower than two minimum wages had lower scores for the psychological domain. Regarding types of diets, pescatarians scored better than the others. Adopting vegetarianism due to aversion/intolerance also had a negative effect on psychological wellbeing, and in contrast, individuals who became vegetarian due to religion/beliefs had higher scores. Having close people adopting the diet also resulted in better scores for the psychological domain.

Domain 3 (social relationships) had higher scores for individuals who tested positive for COVID-19. Results regarding infected family members and BMI were the same as for domains 1 and 2, and the same trend for better scores with higher income levels was also observed for the social domain. The only aspect directly related to vegetarianism that was correlated to domain 3 was having close people also following a vegetarian diet, which resulted in higher scores.

Results for domain 4 (environment) were the same as for the previous domains regarding BMI and educational level. In this case, scores were different depending on the housing location. Individuals living in capitals or metropolitan areas scored better, and those living in urban areas (other locations) had worse scores, with intermediate results for those who declared living in rural areas. The ones with the highest income levels had better scores, with worse results for those with the lowest income levels, following the same trend as for the previous domains. Again, the only feature related to the diet that influenced this domain scores was having close people also following the diet.

## 4. Discussion

Facing a pandemic can negatively affect mental health, wellbeing, social relations and economic aspects, all of which could potentially affect QoL [18,19]. Understanding the impact of the COVID-19 pandemic on the QoL of specific groups is important in order to reveal how each different segment of the population reacted to this global emergency situation.

Two QoL instruments were used in this study. The WHOQOL-BREF is expected to reflect changes in aspects that are more closely related to the impact of contracting a disease (such as COVID-19), as it evaluates more general aspects, related to physical and mental health, social relationships and the environment in which an individual is inserted [14]. Moreover, it has been widely used in studies from several countries, which enables comparisons with different populations or different subgroups of a specific population. On the other hand, general tools might be limited when evaluating QoL related to dietary changes since they may not consider important factors that could influence QoL [20]. Therefore, VEGQOL was also used to measure aspects that are more directly related to vegetarianism and diet changes [13].

Our study showed that individuals who tested positive for COVID-19 had a lower QoL score measured by the VEGQOL. Lower QoL scores in individuals infected by COVID-19 were also found in a study conducted in Brazil from 27 May to 14 August 2020 to evaluate QoL in the general population. A cross-sectional study conducted in Vietnam from 14 February to 2 March 2020 with 3947 adults also revealed that the participants with COVID-19 symptoms were more likely to have depression and lower health-related QoL [21].

Our results must be interpreted with caution, since only 3.8% of the sample declared having tested positive. It is important to acknowledge that, by 14 September 2020 (when the survey was finished), the official number of infected people in Brazil was 4,345,610 [22], which represented 2.1 percent of the estimated Brazilian population in 2020, of 211,755,692 inhabitants [23]. Therefore, we believe that the low prevalence of infected people in our study was simply a relatively close reflection of the national infection rates at that moment, even when considering the possibility of sub notification of COVID-19 cases.

No difference was seen for the WHOQOL-BREF domain scores among infected individuals, except for social relations, in which the score was higher. In contrast with the VEGQOL, which measures specific aspects related to vegetarianism, the WHOQOL would be a more suitable tool to evaluate the impact of the disease on general QoL aspects. It would be expected, for example, that suffering from disease symptoms would negatively affect the WHOQOL scores (especially in the physical and psychological domains). In contrast, we found no negative effects in these domains. A case-control study conducted in six countries with healthcare workers showed that individuals following a plant-based diet had 73 percent lower odds of having severe to moderate COVID-19 [24]. It is possible that individuals who tested positive in our study had only mild symptoms, which were not sufficient to negatively affect QoL during or after the infection.

An online survey conducted in the Liaoning province (China) from 28 January 2020 to 5 February 2020 with 263 adults showed that the majority of participants received increased support from friends (64.6%) and family members (63.9%) [4]. Receiving more support from close and beloved people could explain the potential positive effect on the social relations domain for the individuals who tested positive for COVID-19. It is also possible that the individuals who tested positive were not following the isolation protocols, reducing the negative effects of social distancing. A study conducted in Brazil showed that, during the COVID-19 pandemic, individuals who were not following the social distancing recommendations had better QoL scores [25]. Moreover, individuals who had already been contaminated might have become more flexible toward isolation protocols due to the possibility of immunization after being contaminated.

Having a family member infected by Sars-Cov-2 had negative outcomes for most QoL measured aspects. Concern about the health of beloved ones and the feeling of lack of control and uncertainty can increase anxiety and depression [26], which could have negatively influenced the QoL of the participants.

Participants with normal weight scored better on all the WHOQOL-BREF parameters, as well as in the VEGQOL. It is well known that excess weight is a risk factor for the complications of the COVID-19, increasing the likelihood of having respiratory complications and mortality [27]. Excess weight is also associated with a higher risk for other chronic diseases such as diabetes, cardiovascular disease and cancer, which are also risk factors for increasing COVID-19 lethality [26]. A systematic review [28] showed that, compared to normal-weight adults, those with excess weight had significantly lower physical health-related QoL. A negative influence of BMI on QoL might also be the case for overweight individuals in our study population, who would already have a lower QoL when compared to normal-weight individuals.

The stated income reduction negatively affected only the physical and environmental domains, as well as general items 1 and 2 from WHOQOL-BREF, with no differences in the VEGQOL or in the psychological and social domains. It is possible that income losses would only have a negative impact on the quality of life of those with the lowest incomes, as it has been demonstrated in unemployed people during the pandemic in Brazil [25]. Also, changes in the expenses that would result from planning more economic meals, cooking at home, and not eating out could have helped to counterbalance the income losses [29]. Another possibility is that the QoL of individuals would have been affected equally, regardless of having an income reduction or not.

The educational level of participants was positively correlated with WHOQOL-BREF scores for the physical, psychological, and environmental domains. In addition, in the Brazilian study, higher educational levels were associated with a better perception of QoL during the COVID-19 pandemic [25]. Health literacy was a protective factor for depression and lower health-related QoL in a study conducted in Vietnam during the COVID-19 pandemic [21]. Therefore, having a higher educational level might facilitate the understanding of basic health and COVID-19-related information, positively influencing some of the QoL metrics.

Despite the pandemic, vegetarians still presented good results regarding QoL, with higher scores among vegans. Considering the situation, QoL was expected to be lower compared to previous studies, mainly due to limited access to food variety during the isolation period. However, previous results on QoL among Brazilian vegetarians (*n* = 5014) [13] measured with the VEGQOL were similar to the ones found in this study: vegans had higher scores, followed by vegetarians, then pesco-vegetarians and, lastly, semi-vegetarians (with the lowest scores). Scores found in this study were numerically higher than the ones described in the study before the pandemic: 79.21 ± 10.66 for vegans; 73.13 ± 11.58 for vegetarians; 69.55 ± 12.50 for pesco-vegetarians; and 64.38 ± 12.84 for semi-vegetarians. Scores were also higher among those participants who adopted the diet for a longer time and had close people also adopting a vegetarian diet. Moreover, those adopting the diet for ethical/moral reasons had the highest scores among the different motivations. All these results were reproduced in our study when considering the VEGQOL scores [13]. Since social interactions reduced drastically during the pandemic, it is possible that some vegetarians would perceive an increase in QoL, as they would suffer less with stigmatization and rejection by omnivores, which often occurs in social situations [30].

It is also important to acknowledge that many other factors related to the pandemic could influence individuals’ QoL, besides COVID-19 infection or income reduction. A pandemic outbreak can cause panic and mental stress, as well as a constant worry of getting infected [19]. The risk of infection can vary greatly, affecting individuals in very different ways. Some of the factors that influence the risk are population density, household size, and social distancing levels. Social distancing rules might be challenging to follow for people whose jobs cannot be conducted remotely. Moreover, individuals who live in smaller and crowded houses might face more difficulties related to social distancing regulation [31].

The way that the Brazilian government politically handled the COVID-19 pandemic might have influenced the results as well. By comparing the SARS-Cov-2 to a common flu virus and not taking strict measures to control its spread, the president might have created a false idea that the situation was not as serious as it really was. Hence, it could be speculated that a considerable parcel of the Brazilian population did not feel worried or endangered by the situation. Future research with more segmented analysis focused on different variables could reveal more details of how the COVID-19 pandemic affected vegetarians, as well as the general population.

Using a convenience sample is a limitation of this study. However, considering that vegetarians represent only 14% of the Brazilian population [32], using random sampling would not allow us to achieve a sufficient number of individuals to conduct all stratified analyses. Moreover, using a convenience sample and conducting an online survey made it possible for us to include enough individuals who tested positive for COVID-19. Only aspects related to COVID-19 infection and income reduction were included as variables, limiting the interpretation of the indicators obtained in this study. Future research is needed in order to explore other potential factors influencing QoL.

Our sample consisted mainly of females and young-age individuals, which can also be considered a limitation, as it is not possible to extrapolate data to all vegetarians in Brazil. However, other studies have already demonstrated that most vegetarians are women and younger than omnivores or semi-vegetarians [31,33]. Finally, the length of time during which the research was conducted (from July to October 2020) could influence the results, due to the very dynamic characteristic of the COVID-19 pandemic. However, since the proportion of infected participants was low (3.8 percent), conducting the study for a shorter period could have impaired some of the analyses, as the number of responses could have been too low to enable comparisons.

## 5. Conclusions

This is the first study on vegetarians’ QoL during the pandemic. Only a minor proportion of the study sample (3.8%) had already tested positive for COVID-19. On those individuals, QoL was lower only in the VEGQOL score, representing more specific aspects of QoL related to vegetarianism. However, QoL was lower among the participants who declared that Sars-Cov-2 had already infected a family member, not only when measured by the VEGQOL but also in three out of the four WHOQOL-BREF domains, indicating that the infection of a family member might have more negative impacts on QoL than having had the infection themselves. Moreover, an income drop affected QoL only partially. VEGQOL scores were better for vegans, for those who followed a vegetarian diet for longer, and for individuals who had close people also following a vegetarian diet.

More studies including other variables and evaluating the impact of the COVID-19 pandemic in other population groups and other time frames are necessary to help create a clearer picture of the impact of the pandemic on QoL. Studying how specific population groups such as vegetarians react to and are influenced by such conditions contributes to the generation of relevant data that could be used to support healthcare and other policies in the future.

## Figures and Tables

**Table 1:** Characteristics of the study sample.

Characteristic	Category	Respondents (*n* = 1282)	
		Number	Percentage
Tested positive for COVID-19	No	1231	96.0%
	Yes	49	3.8%
	Not informed	2	0.2%
Family member infected	No	768	59.9%
	Yes (do not live with me)	418	32.6%
	Yes (lives with me)	93	7.3%
	Not informed	3	0.2%
Reduction of income	No	686	53.5%
	Yes	594	46.3%
	Not informed	2	0.2%
Gender	Male	168	13.1%
	Female	1114	86.9%
Age	18–24	607	47.4%
	25–29	267	20.8%
	30–39	327	25.5%
	40–49	59	4.6%
	50–59	19	1.5%
	60 or more	3	0.2%
Housing location	Capital or metropolitan area	974	76.0%
	Urban area (other cities)	284	22.2%
	Rural area	24	1.9%
Average income ^(1)^	Less than two minimum wages	126	9.8%
	Between two and five minimum wages	316	24.6%
	Between five and ten minimum wages	343	26.8%
	Between ten and twenty minimum wages	273	21.3%
	Above twenty minimum wages	134	10.4%
	Not informed	90	7.0%
Educational level	No education	0	0%
	Elementary School, incomplete	1	0.1%
	Elementary School, complete	3	0.2%
	High School, incomplete	31	2.4%
	High School, complete	175	13.7%
	University level, incomplete	415	32.4%
	University level, complete	657	51.2%
BMI ^(2)^	<18.5 kg/m^2^	90	7.0%
	18.5 a 24.9 kg/m^2^	847	66.1%
	> 24.9 kg/m^2^	342	26.7%
	Not informed	3	0.2%
Type of diet	Vegan	392	30.6%
	Ovolacto-vegetarian	614	47.9%
	Pescatarian	124	9.7%
	Flexitarian	152	11.8%
Time adopting the diet	Less than 1 year	371	29.0%
	Between 1 and 5 years	635	49.5%
	More than 5 years	276	21.5%
Main motivation	Ethic/moral	722	56.3%
	Personal health	124	9.7%
	Religion / beliefs	35	2.7%
	Environmental impact	251	19.6%
	Aversion/intolerance	64	5.0%
	Others	86	6.7%
Close people also	No	336	26.2%
adopting the diet	Yes	946	73.8%

^(1)^ One minimal wage is equivalent to R$1045.00 or US$232.74 (in 2020). ^(2)^ Source: [17].

**Table 2:** Mean and standard deviation from VEGQOL and WHOQOL-BREF results, according to sociodemographic characteristics (*n* = 1282).

Characteristic	VEGQOL					Whoqol	
		Q1	Q2	D1 Physical Health	D2 Psychological Wellbeing	D3 Social Relationships	D4 Environment
Tested positive for COVID-19							
No	75.45 (11.43) ^a^	4.26 (0.66) ^a^	3.86 (0.87) ^a^	3.80 (0.63) ^a^	3.48 (0.64) ^a^	3.45 (0.79) ^a^	3.78 (0.60) ^a^
Yes	71.51 (13.02) ^b^	4.22 (0.71) ^a^	4.08 (0.70) ^a^	3.84 (0.64) ^a^	3.57 (0.65) ^a^	3.69 (0.75) ^b^	3.81 (0.55) ^a^
*p* ^(1)^	0.019 ^(1)^	0.800 ^(3)^	0.095 ^(3)^	0.646 ^(1)^	0.331 ^(1)^	0.042 ^(1)^	0.690 ^(1)^
Family member infected							
No	76.03 (11.18) ^a^	4.29 (0.66) ^a^	3.92 (0.86) ^a^	3.84 (0.62) ^a^	3.53 (0.64) ^a^	3.51 (0.80) ^a^	3.81 (0.59) ^a^
Yes (do not live with me)	74.30 (11.87) ^b^	4.21 (0.68) ^a^	3.77 (0.87) ^b^	3.73 (0.65) ^b^	3.41 (0.63) ^b^	3.36 (0.79) ^b^	3.74 (0.61) ^a^
Yes (lives with me)	73.64 (12.21) ^ab^	4.30 (0.64) ^a^	3.89 (0.94) ^ab^	3.82 (0.59) ^ab^	3.48 (0.65) ^ab^	3.51 (0.74) ^ab^	3.79 (0.62) ^a^
*p* ^(2)^	0.016 ^(2)^	0.176 ^(4)^	0.010 ^(4)^	0.019 ^(2)^	0.008 ^(2)^	0.005 ^(2)^	0.147 ^(2)^
Gender							
Male	73.83 (11.43) ^a^	4.22 (0.70) ^a^	3.84 (0.92) ^a^	3.93 (0.63) ^a^	3.56 (0.66) ^a^	3.39 (0.88) ^a^	3.73 (0.60) ^a^
Female	75.51 (11.51) ^a^	4.27 (0.66) ^a^	3.87 (0.86) ^a^	3.78 (0.63) ^b^	3.48 (0.64) ^b^	3.47 (0.78) ^a^	3.79 (0.60) ^a^
*p* ^(1)^	0.078 ^(1)^	0.487 ^(3)^	0.862 ^(3)^	0.003 ^(1)^	0.013 ^(1)^	0.215 ^(1)^	0.231 ^(1)^
Age							
Below 40 years old	75.23 (11.47) ^a^	4.26 (0.67) ^a^	3.86 (0.87) ^a^	3.79 (0.63) ^a^	3.47 (0.65) ^a^	3.46 (0.80) ^a^	3.78 (0.60) ^a^
40 years old or more	76.23 (12.13) ^a^	4.30 (0.58) ^a^	3.99 (0.90) ^a^	3.90 (0.62) ^a^	3.73 (0.51) ^b^	3.43 (0.70) ^a^	3.75 (0.51) ^a^
*p* ^(1)^	0.445 ^(1)^	0.875 ^(3)^	0.097 ^(3)^	0.140 ^(1)^	<0.001 ^(1)^	0.694 ^(1)^	0.643 ^(1)^
BMI ^(4)^							
<18.5 kg/m^2^	75.83 (11.11) ^ab^	4.21 (0.68) ^ab^	3.83 (0.77) ^ab^	3.70 (0.68) ^ab^	3.42 (0.65) ^ab^	3.41 (0.86) ^ab^	3.73 (0.70) ^ab^
18.5 a 24.9 kg/m^2^	75.83 (11.31) ^a^	4.32 (0.63) ^a^	4.00 (0.80) ^a^	3.85 (0.60) ^a^	3.54 (0.61) ^a^	3.51 (0.77) ^a^	3.82 (0.57) ^a^
>24.9 kg/m^2^	73.73 (11.99) ^b^	4.12 (0.73) ^b^	3.54 (0.96) ^b^	3.69 (0.68) ^b^	3.37 (0.68) ^b^	3.36 (0.81) ^b^	3.69 (0.62) ^b^
*p* ^(2)^	0.015 ^(2)^	<0.001 ^(4)^	<0.001 ^(4)^	<0.001 ^(2)^	<0.001 ^(2)^	0.015 ^(2)^	0.002 ^(2)^
Educational level							
High school or lower	74.68 (10.92) ^a^	4.28 (0.64) ^a^	3.80 (0.87) ^a^	3.65 (0.66) ^a^	3.33 (0.68) ^a^	3.38 (0.76) ^a^	3.70 (0.66) ^a^
Graduation or higher	75.41 (11.62) ^a^	4.26 (0.67) ^a^	3.88 (0.87) ^a^	3.83 (0.62) ^b^	3.52 (0.63) ^b^	3.48 (0.80) ^a^	3.80 (0.58) ^b^
*p* ^(2)^	0.401 ^(1)^	0.768 ^(3)^	0.144 ^(3)^	<0.001 ^(1)^	<0.001 ^(1)^	0.107 ^(1)^	0.044 ^(1)^
Housing location							
Capital or metropolitan area	75.13 (11.60) ^a^	4.28 (0.67) ^a^	3.87 (0.89) ^a^	3.81 (0.63) ^a^	3.50 (0.64) ^a^	3.48 (0.79) ^a^	3.82 (0.60) ^a^
Urban area (other cities)	75.41 (11.18) ^a^	4.21 (0.66) ^a^	3.85 (0.82) ^a^	3.75 (0.63) ^a^	3.44 (0.65) ^a^	3.40 (0.79) ^a^	3.66 (0.58) ^b^
Rural area	80.37 (10.91) ^a^	4.25 (0.74) ^a^	3.79 (0.59) ^a^	4.02 (0.56) ^a^	3.54 (0.61) ^a^	3.44 (0.73) ^a^	3.74 (0.56) ^ab^
*p* ^(2)^	0.087 ^(2)^	0.220 ^(4)^	0.459 ^(4)^	0.108 ^(2)^	0.352 ^(2)^	0.386 ^(2)^	<0.001 ^(2)^
Average income ^(3)^							
<2 minimum wages	76.56 (11.19) ^a^	4.00 (0.70) ^a^	3.60 (0.95) ^a^	3.60 (0.64) ^a^	3.33 (0.68) ^a^	3.29 (0.81) ^a^	3.23 (0.64) ^a^
2–5 minimum wages	76.09 (11.46) ^a^	4.14 (0.69) ^ab^	3.85 (0.85) ^ab^	3.76 (0.61) ^ab^	3.44 (0.64) ^ab^	3.42 (0.85) ^ab^	3.53 (0.58) ^b^
5–10 minimum wages	75.24 (11.44) ^a^	4.28 (0.65) ^bc^	3.83 (0.89) ^ab^	3.75 (0.64) ^a^	3.53 (0.63) ^b^	3.50 (0.75) ^ab^	3.85 (0.52) ^c^
10–20 minimum wages	74.64 (11.65) ^a^	4.35 (0.66) ^c^	3.95 (0.87) ^bc^	3.93 (0.61) ^bc^	3.56 (0.62) ^b^	3.47 (0.76) ^ab^	4.03 (0.47)^d^
> 20 minimum wages	74.11 (11.98) ^a^	4.57 (0.54)^d^	4.10 (0.78) ^c^	3.94 (0.60) ^c^	3.58 (0.62) ^b^	3.61 (0.71) ^b^	4.22 (0.36)^e^
*p* ^(2)^	0.259 ^(2)^	<0.001 ^(4)^	<0.001 ^(4)^	<0.001 ^(2)^	0.002 ^(2)^	0.012 ^(2)^	<0.001 ^(2)^
Reduction of income							
No	75.38 (10.84) ^a^	4.34 (0.64) ^a^	3.92 (0.84) ^a^	3.85 (0.61) ^a^	3.50 (0.63) ^a^	3.47 (0.79) ^a^	3.92 (0.53) ^a^
Yes	75.23 (12.24) ^a^	4.18 (0.68) ^b^	3.81 (0.89) ^b^	3.75 (0.65) ^b^	3.47 (0.66) ^a^	3.45 (0.80) ^a^	3.62 (0.63) ^b^
*p* ^(1)^	0.827 ^(1)^	<0.001 ^(3)^	0.022 ^(3)^	0.005 ^(1)^	0.423 ^(1)^	0.544 ^(1)^	0.000 ^(1)^
Type of diet							
Vegan	80.70 (10.07) ^a^	4.33 (0.63) ^a^	4.10 (0.76) ^a^	3.90 (0.64) ^a^	3.56 (0.67) ^ab^	3.51 (0.76) ^a^	3.76 (0.58) ^a^
Ovolacto-vegetarian	75.00 (10.37) ^b^	4.24 (0.67) ^ab^	3.83 (0.86) ^b^	3.75 (0.62) ^bc^	3.43 (0.65) ^a^	3.43 (0.81) ^a^	3.77 (0.61) ^a^
Pescatarian	70.46 (11.57) ^c^	4.38 (0.63) ^a^	3.81 (0.86) ^b^	3.85 (0.55) ^ab^	3.60 (0.57) ^b^	3.55 (0.77) ^a^	3.94 (0.53) ^b^
Flexitarian	66.43 (11.86)^d^	4.09 (0.74) ^b^	3.47 (1.00) ^c^	3.69 (0.64) ^c^	3.41 (0.56) ^a^	3.39 (0.81) ^a^	3.76 (0.64) ^a^
*p* ^(2)^	<0.001 ^(2)^	<0.001 ^(4)^	<0.001 ^(4)^	<0.001 ^(2)^	0.001 ^(2)^	0.164 ^(2)^	0.019 ^(2)^
Time adopting diet							
Less than 1 year	71.54 (12.04) ^a^	4.23 (0.66) ^a^	3.80 (0.88) ^a^	3.77 (0.63) ^a^	3.47 (0.62) ^a^	3.44 (0.78) ^a^	3.76 (0.59) ^a^
Between 1 and 5 years	76.41 (10.85) ^b^	4.30 (0.64) ^a^	3.88 (0.85) ^a^	3.79 (0.62) ^a^	3.47 (0.65) ^a^	3.47 (0.79) ^a^	3.79 (0.60) ^a^
More than 5 years	77.75 (11.07) ^b^	4.22 (0.73) ^a^	3.93 (0.88) ^a^	3.86 (0.64) ^a^	3.54 (0.66) ^a^	3.47 (0.81) ^a^	3.79 (0.60) ^a^
*p* ^(2)^	<0.001 ^(2)^	0.252	0.107	0.143 ^(2)^	0.234 ^(2)^	0.759 ^(2)^	0.605 ^(2)^
Main motivation							
Ethic/moral	76.40 (11.24) ^a^	4.27 (0.66) ^a^	3.90 (0.87) ^a^	3.81 (0.63) ^ab^	3.46 (0.65) ^ab^	3.47 (0.77) ^a^	3.77 (0.59) ^a^
Personal health	74.64 (11.33) ^ab^	4.23 (0.63) ^a^	3.86 (0.88) ^a^	3.90 (0.61) ^ab^	3.62 (0.57) ^ab^	3.50 (0.79) ^a^	3.80 (0.70) ^a^
Religion/beliefs	74.95 (12.29) ^ab^	4.20 (0.53) ^a^	3.86 (0.81) ^a^	3.80 (0.56) ^ab^	3.71 (0.59) ^a^	3.67 (0.78) ^a^	3.60 (0.54) ^a^
Environmental impact	73.28 (11.64) ^b^	4.31 (0.66) ^a^	3.83 (0.86) ^a^	3.73 (0.62) ^ab^	3.47 (0.64) ^ab^	3.43 (0.83) ^a^	3.81 (0.55) ^a^
Aversion/intolerance	71.60 (13.33) ^b^	4.09 (0.87) ^a^	3.67 (0.94) ^a^	3.63 (0.65) ^a^	3.39 (0.73) ^b^	3.23 (0.88) ^a^	3.71 (0.72) ^a^
Others	75.63 (10.68) ^ab^	4.28 (0.68) ^a^	3.81 (0.85) ^a^	3.91 (0.58) ^b^	3.55 (0.63) ^ab^	3.52 (0.78) ^a^	3.91 (0.49) ^a^
*p* ^(2)^	0.001 ^(2)^	0.472 ^(4)^	0.335 ^(4)^	0.017 ^(2)^	0.017 ^(2)^	0.106 ^(2)^	0.108 ^(2)^
Close people also adopting the diet							
No	72.02 (12.62) ^a^	4.14 (0.70) ^a^	3.78 (0.91) ^a^	3.73 (0.64) ^a^	3.38 (0.68) ^a^	3.33 (0.83) ^a^	3.65 (0.63) ^a^
Yes	76.45 (10.86) ^b^	4.31 (0.65) ^b^	3.90 (0.85) ^a^	3.82 (0.62) ^b^	3.52 (0.62) ^b^	3.51 (0.77) ^b^	3.83 (0.58) ^b^
*p* ^(1)^	<0.001 ^(1)^	<0.001 ^(3)^	0.053 ^(3)^	0.029 ^(1)^	0.001 ^(1)^	<0.001 ^(1)^	<0.001 ^(1)^

^(1)^ Independent Student *t*-test. ^(2)^ Anova with Tukey’s post-hoc tests. ^(3)^ Mann-Whitney test. ^(4)^ Kruskall-Wallis test with Bonferroni’s post-hoc tests. a, b, c Categories with same letter do not differ significantly (*p* > 0.05). One minimal wage is equivalent to R$1045.00 or US$232.74 (in 2020). Source: WHO/ Body Mass Index [17]. Categories with the same letters do not differ significantly (*p* > 0.05).

## Data Availability

Data sharing is not applicable to this article.
